# Remarkable Case of Masked Ague

**Published:** 1829-10-01

**Authors:** 


### HOPITAL ST. ANTOINE.

### Curious Case of Masked Ague.

A young female, aged 16 years, had had only two or three appearances of menstruation, and reported that she was subject to general malaise for many years. During the year preceding her entrance into the Hôpital St. Antoine (on the 20th May, 1828,) the malady of which she complained assumed the intermittent type. The accessions were at first every three weeks—afterwards once a fortnight; but, during the last two months, they occurred regularly every eighth day. The precursory phenomenon was a swelling of one hand, generally the right—but this precursor did not invariably take place. The swelling usually lasted two days, and, as this subsided, colick, head-ache, and fever supervened, lasting about 24 hours. The paroxysm came on with a violent rigor, of two or three hours' continuance, then fever, and, ultimately, perspiration. Occasionally the sweating stage did not take place. It was remarked that there was no thirst during the paroxysm; but it came on very urgently after the accession was over. Vomiting was a very common symptom during the attack, and the colicky pains, with head-ache, continued for two days after each. The epigastric region was very sensible and painful on pressure. The malady was watched during a month at the hospital, and four paroxysms were carefully noted. The sulphate of quinine was then administered, and stopped the complaint.—Bibliothèque Médicale.

It is remarked that the complaint seemed to be hereditary in the family; as the mother and five or six of the sisters and brothers were subject to it. We should suppose that the cause was rather in the place where they lived, than in any thing hereditary. It is an instance of one of the many anomalous forms which ague will assume, and by which it often eludes the vigilance of the practitioner.

